# The Role of O-GlcNAcylation in Immune Cell Activation

**DOI:** 10.3389/fendo.2021.596617

**Published:** 2021-04-27

**Authors:** Amy Qiang, Chad Slawson, Patrick E. Fields

**Affiliations:** ^1^ Pathology, University of Kansas Medical Center, Kansas City, KS, United States; ^2^ Biochemistry, University of Kansas Medical Center, Kansas City, KS, United States

**Keywords:** inflammation, T cell, macrophage, O-GlcNAc, cytokine release syndrome (CRS)

## Abstract

O-GlcNAcylation is a dynamic post-translational modification where the sugar, O-linked β-N-acetylglucosamine (O-GlcNAc) is added to or removed from various cytoplasmic, nuclear, and mitochondrial proteins. This modification is regulated by only two enzymes: O-GlcNAc transferase (OGT), which adds O-GlcNAc, and O-GlcNAcase (OGA), which removes the sugar from proteins. O-GlcNAcylation is integral to maintaining normal cellular function, especially in processes such as nutrient sensing, metabolism, transcription, and growth and development of the cell. Aberrant O-GlcNAcylation has been associated with a number of pathological conditions, including, neurodegenerative diseases, cancer, diabetes, and obesity. However, the role of O-GlcNAcylation in immune cell growth/proliferation, or other immune responses, is currently incompletely understood. In this review, we highlight the effects of O-GlcNAcylation on certain cells of the immune system, especially those involved in pro-inflammatory responses associated with diabetes and obesity.

## Introduction

The immune system is a complex network of cells and proteins that has evolved to help the body eradicate viral and bacterial pathogens. The pro-inflammatory responses of both the innate and adaptive immune systems work in tandem to facilitate this activity. Inflammation caused by the immune system in response to microbial infection and tissue damage provides protection against future infections and sets up long-term adaptive immunity against these pathogens. Adaptive immune memory cells trigger innate inflammatory immune responses to previously encountered pathogens, initiating a quicker response to bolster the defenses against infection. Both the innate and adaptive immune systems are pro-inflammatory when activated; however, chronic inflammatory response contributes to numerous disease states while the mechanisms by which these responses are initiated are not fully understood. One modification that plays a major role in initiating the pro-inflammatory states of the immune system is O-GlcNAcylation, and the potential mechanisms by which it does so are discussed below.

## T Cell Activation Induces a Metabolic Shift in Immune Cells

Naive CD4^+^ T cells can differentiate into several classes of activated T cells. Antigen-presenting cells (APC) with major histocompatibility complex II (MHC II) present antigenic peptides on its surface to activate naive T cells if the T cell receptor recognizes the antigen, and if a second signal from a co-stimulatory molecule like CD28 is also present (1, 2). The T cell then becomes activated to respond specifically to that antigen and proliferates rapidly to eliminate it (3). In order to maintain the massive proliferation of the T cells, a complete metabolic switch in adaptive immune cells is needed.

The metabolites needed to sustain this response rely on aerobic glycolysis and glutaminolysis rather than oxidative phosphorylation. This causes a sharp increase of various metabolites that promote cell growth and differentiation. Early studies demonstrated a sharp increase of glucose into activated T cells (4). In addition to glucose, activated T cells also require increased amounts of amino acids, such as glutamine (5). Glutamine uptake is increased in activated T cells ten times more than any other amino acid, underscoring the dependence on this amino acid as an energy source for these cells (6). In addition to its role in increased protein translation, glutamine serves as a source of oxaloacetate (OAA) in the TCA cycle, which produces citrate, that is transported out of the mitochondria and then converted to acetyl-CoA (7). Acetyl-CoA is vital for fatty acid and cholesterol synthesis (8); thus, increased glutamine levels result in increased production of acetyl CoA, allowing for greater fatty acid synthesis during the metabolic shift induced by activated T cells. Notably, the metabolites produced during these processes serve as substrates in the hexosamine biosynthetic pathway (HBP), enabling the production of UDP-GlcNAc, a necessary substrate for protein O-GlcNAcylation (9).

## O-GlcNAcylation Regulates T Cell Activation and Differentiation

O-GlcNAcylation is a post-translational protein modification resulting from the enzymatic addition of a single, O-linked β-N-acetylglucosamine (O-GlcNAc) molecule to the serine or threonine residues of a protein. This modification can cycle on and off of many cellular proteins found in the cytoplasm, nucleus, and mitochondria. The two enzymes that regulate this dynamic cycling are O-GlcNAc transferase (OGT), which adds the sugar to proteins, and O-GlcNAcase (OGA), which removes the sugar from proteins (10).

Multiple studies have provided support for the idea that the dynamic cycling of O-GlcNAc plays an important role in the activation and regulation of T cells. First, lymphocyte activation during thymic development results in a rapid *increase* in levels of O-GlcNAc on nuclear proteins and a coincident *decrease* in levels on cytosolic proteins, suggesting a regulatory role in early T cell development (11). Second, the downregulation of OGT *via* siRNA-mediated knockdown leads to impaired IL-2 production, which affects the proliferative response of T cells (12). Consistent with this, activation of primary human T cells through the T cell receptor (TCR) leads to increased O-GlcNAc levels and elevation in the expression of OGT, but not OGA, suggesting that the addition of O-GlcNAc, but not its removal, is important for T cell activation and cytokine production (13). This study also identified several proximal O-GlcNAc substrates, such as ZAP-70, SHIP1, and LCK, which are directly involved in immune cell signaling *via* antigenic stimulation of the TCR. Increased OGT in mouse adipocytes promotes the expression of leptin (14). The adipose microenvironment is rich in CD4^+^ T cells, with leptin signaling being an important regulator of T cell growth and function (15). These findings demonstrate the importance of O-GlcNAc early in the TCR pathway and its subsequent role in regulating T cell activation.

Following TCR activation, naive CD4^+^ T cells can be differentiated into any one of a variety of effector lineages, depending on the microenvironment in which the cell differentiates and the proximal signals the cell receives (e.g. cytokines, APC ligands, etc.) These effector lineages include T helper (Th)1, Th2, Th17, and regulatory T cells (Tregs). Each of these effector cell types perform distinct functions. Th1, Th2, and Th17 cells are responsible for the elimination of various pathogens, while Tregs are responsible for the reduction of the inflammatory immune response. Interestingly, O-GlcNAcylation is necessary for both the differentiation and homeostasis of both Th17 cells and Tregs (16, 17), outlining two opposing functions in immune cell signaling. The production of IL-17A, a major pro-inflammatory cytokine secreted by Th17 cells, is significantly increased in response to treatment of splenic CD4^+^ T cells with Thiamet G (TMG), a highly selective OGA inhibitor (16). TMG treatment also increased the binding of RAR-related orphan receptor gamma (RORγt) to the IL-17 promoter. Another pro-inflammatory marker of Th17 function, the IL-23 receptor, increased in response to TMG treatment. In adipose tissue, saturated fatty acids released by the adipocytes promotes differentiation of Th17 cells from naïve CD4^+^ T cells and increases the production of both IL-17 and IFNγ (18). Taken together, the elevation of O-GlcNAcylation promotes IL-17 production and Th17 differentiation. On the other hand, O-GlcNAcylation is also needed to stabilize Tregs by regulating the transcription factor Forkhead box P3 (FOXP3) and activating STAT5 (17). The varying roles of O-GlcNAcylation in T cell differentiation demonstrates its importance as a regulator of immune responses and T cell homeostasis.

## O-GlcNAcylation Causes Other Pro-Inflammatory Immune Responses

As mentioned, the HBP produces UDP-GlcNAc, which is the substrate utilized by OGT to O-GlcNAcylate proteins. The amount of UDP-GlcNAc produced by the HBP relies on the availability of glucose in a cell, with 2-3% of the glucose entering a cell continuing down this pathway (19). Under hyperglycemic conditions, such as in diabetes or obesity, there is increased glucose flux through the HBP, consequently producing more UDP-GlcNAc which then elevates O-GlcNAcylation levels.

Higher levels of OGT in adipose tissue are also linked to pro-inflammatory signaling in diabetes and hyperleptinemia, again illustrating the role OGT plays in regulating immune cells in adipose tissue (14). O-GlcNAcylation of the transcription factor nuclear factor kappa-light-chain-enhancer of activated B cells (NF-κB) under hyperglycemic conditions increases transcriptional activity and decreases binding to IκBα, a regulatory protein that inhibits NF-κB complex activity (20). NF-κB plays an important role in T cell function and development, especially in the subset Th17 cells, which produce pro-inflammatory cytokines such as IL-17A, IL-17F, IL-21, and IL-22 (21). Thus, over-stimulation of the NF-κB signaling pathway, such as during increased O-GlcNAcylation in hyperglycemia, has pro-inflammatory effects.

Other transcription factors that are modified by O-GlcNAcylation include nuclear factor of activated T-cells (NFAT), which is crucial for the function and differentiation of T helper cells, such as Th1, Th2, and Th17 cells (22), and STAT3, which is activated by numerous cytokines and growth factors, including IL-6, a pro-inflammatory cytokine whose dysregulation is related to the development of colorectal cancer (23). O-GlcNAcylation induced by fibroblast growth factor 23 results in increased activation of NFAT and secretion of IL-6 in the regulation of airway inflammation in human epithelial bronchial cells (24). In addition, under hyperglycemic conditions, higher levels of O-GlcNAcylation led to changes in specific STAT3 sites of diabetic rat retinas (25). STAT3 O-GlcNAcylation also negatively regulats its phosphorylation and the production of IL-10, exacerbating inflammation and inflammation-driven tumorigenesis in colon macrophages (26). Taken together, these findings illustrate the necessity of O-GlcNAcylation in promoting inflammation as part of the initial immune response.

## O-GlcNAcylation Promotes Opposing Effects in Macrophages

M1 macrophages promote inflammation by secreting pro-inflammatory cytokines and chemokines as part of the initial immune response, such as IL-6, IL-10, and TNFα. On the other hand, M2 macrophages are important in wound healing and tissue repair, secreting anti-inflammatory cytokines like IL-10 and TGF-β. Changes in O-GlcNAcylation affects the polarization of both kinds of macrophages, suggesting a regulatory role in these innate immune cells.

The aggregation of pro-inflammatory macrophages accompanies conditions such as diabetes and obesity. O-GlcNAcylation has an essential function in promoting antiviral innate immunity, as human and murine cells with OGT deficiencies reported defective antiviral responses upon vesicular stomach virus (VSV) challenge (27, 28). Cells with normal OGT levels demonstrate an increase in the HBP upon VSV challenge, elevating O-GlcNAcylation of mitochondrial antiviral-signaling protein (MAVS) to promote the innate immune system. Thus, O-GlcNAcylation caused by VSV challenge increases the function of M1 macrophages, boosting the pro-inflammatory immune response.

Increased HBP activity is also a hallmark of the polarization of M2 macrophages. The N-glycosylation pathway, which requires uridine diphosphate N-acetylglucosamine (UDP-GlcNAc) as a sugar donor, is essential for the expression of M2 activation markers Relmα, CD206, and CD301 (29). Even though N-glycosylation has different functions than O-GlcNAcylation, both share the substrate UDP-GlcNAc, suggesting that changes in O-GlcNAcylation may affect N-glycosylation levels (30) and thus M2 macrophage polarization. For example, another study found that depletion of OGT in the human macrophage cell line THP-1 adversely affected M2 polarization, but M1 genes were upregulated (31). OGT also inhibits the pro-inflammatory activation of macrophages by suppressing the phosphorylation of S6 kinase β-1, suppressing mTORC1 signaling, which prevents pro-inflammatory gene transcription (32). O-GlcNAcylation is therefore shown to be an essential regulator of macrophage function, affecting both the initial inflammatory response of M1 macrophages and the anti-inflammatory response of M2 macrophages.

## OGT Exacerbates Cytokine Release Syndrome in Hyperglycemic Conditions

**Figure 1:**
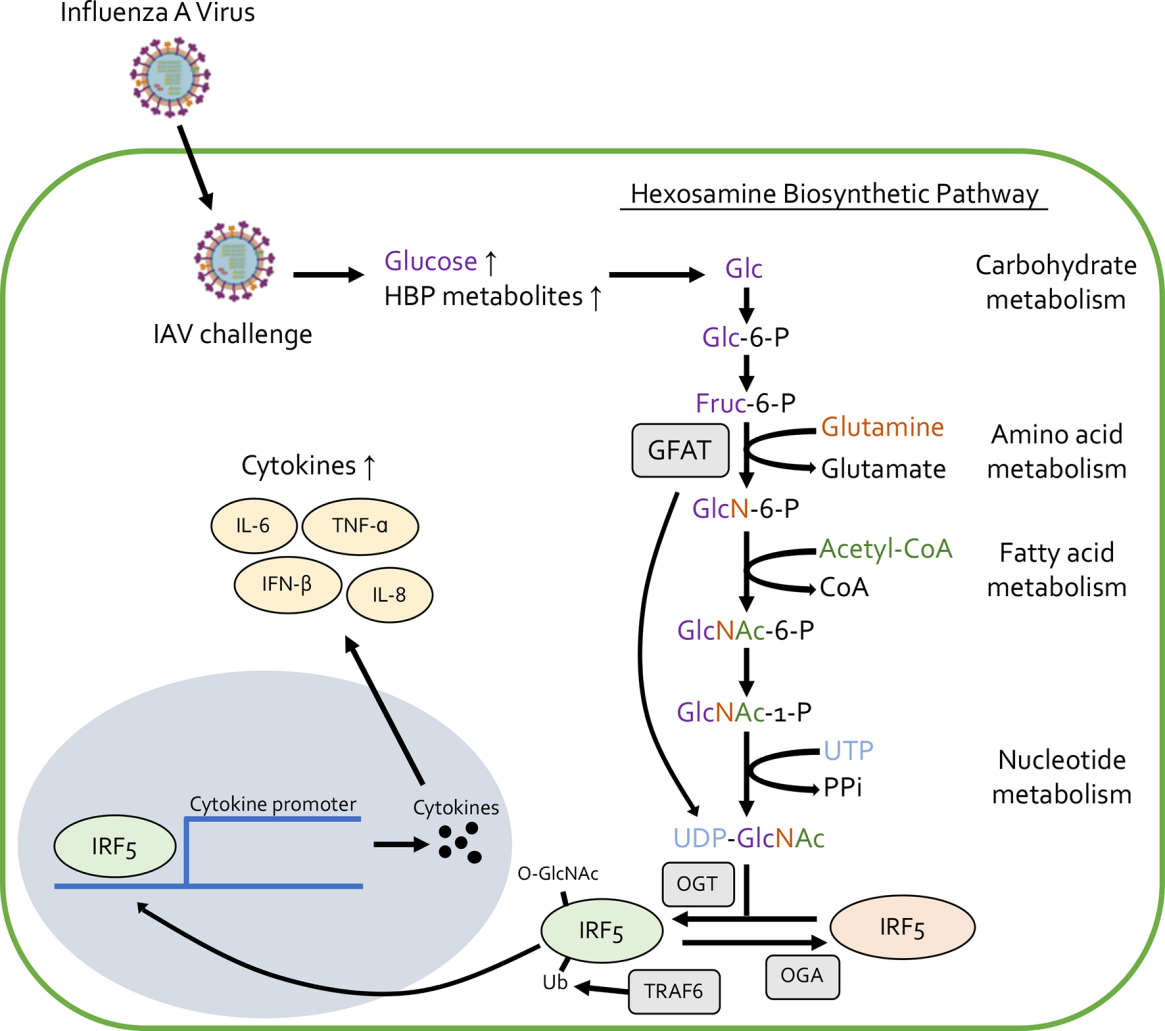
Influenza A virus challenge increases the availability of glucose and other metabolites involved in the hexosamine biosynthetic pathway (HBP), leading to increased synthesis of UDP-GlcNAc and protein O-GlcNAcylation. O-GlcNAcylation of IRF5 causes translocation from the cytoplasm to the nucleus and elevates the production of pro-inflammatory cytokines like IL-6, IL-8, IFN-β, and TNF-α.

Cytokine release syndrome (CRS) is a systemic inflammatory response that can be induced by a number of factors, including severe viral infection (33). Recently, OGT was found to induce CRS caused by influenza A virus (IAV) challenge (34). IAV infection increases the amount of many metabolites involved in both glycolysis and the HBP, leading to more O-GlcNAcylation due to the increased availability of UDP-GlcNAc (**Figure 1**). OGT interacts with interferon regulatory factor 5 (IRF5) to mediate this IAV-induced cytokine storm. Furthermore, blood samples from patients with IAV showed higher blood glucose levels and higher expression of inflammatory cytokines like IL-6 and IL-8. This study links high blood glucose levels (thus increased O-GlcNAcylation), with inflammatory cytokine production upon IAV challenge (**Figure 1**). Future studies could focus on the role of O-GlcNAc in other CRS related diseases to further elucidate its role in causing this pro-inflammatory condition.

In fact, CRS is often present in severe cases of COVID-19. Patients with severe cases had higher levels of pro-inflammatory cytokines like IL-6, IL-10, and TNFα (35). In addition, patients with pre-existing conditions, such as diabetes, are at higher risk for a rapid and more severe progression of COVID-19 and are more susceptible to the development of a cytokine storm (36). O-GlcNAcylation is increased in hyperglycemic conditions, including diabetes, suggesting that O-GlcNAcylation may play a role in mediating CRS in severe cases of COVID-19. A link between O-GlcNAc and SARS-CoV-2 was found, as prolonged TMG treatment in tissue culture cells decreased expression of the gene *SARS*, which encodes the ACE2 receptor (bound by SARS-CoV-2) (37). Further exploration of the relationship between O-GlcNAcylation and the gene *SARS*, as well as its involvement in the onset of the pro-inflammatory cytokine storm, could lead to the development of novel immunotherapies for combating COVID-19.

O-GlcNAc has been the most widely studied in T cells, but there is a burgeoning interest in its effects on other cells in the immune system. Initial studies have found varying roles, both pro-inflammatory and anti-inflammatory, for this post-translational modification. But the specific mechanisms and reasons for its variations have yet to be deeply explored. Further studies are needed in order to more clearly understand the active role that this nutrient-sensitive modification has in the immune system.

## Author Contributions

AQ-Principal author of the current manuscript. CS-significant contributor-edited and co-wrote the manuscript. PF-significant contributor-edited and co-wrote the manuscript. All authors contributed to the article and approved the submitted version.

## Funding

CS was supported by the National Institute on Aging: grant# R01AG064227.

## Conflict of Interest

The authors declare that the research was conducted in the absence of any commercial or financial relationships that could be construed as a potential conflict of interest.
